# Understanding Gender Equality Policy and Practice Gaps Through the Lens of Organisational Justice: Development of an Employee Alignment Model

**DOI:** 10.3389/fsoc.2021.681086

**Published:** 2021-11-30

**Authors:** Chrissi McCarthy, Sarah Barnard, Derek Thomson, Andrew Dainty

**Affiliations:** ^1^ Architecture, Building and Civil engineering, Loughborough University, Loughborough, United Kingdom; ^2^ School of business and economics, Loughborough University, Loughborough, United Kingdom; ^3^ Constructing Equality Limited, Liverpool, United Kingdom

**Keywords:** gender equality, organisational justice, case study method, fairness, behavioural equality

## Abstract

Policies and actions to address gender inequalities are widespread across a range of institutional and organisational contexts. Concerns have been raised about the efficacy and impacts of such measures in the absence of sustained evaluation of these activities. It has been proposed that important contextual factors may propel or inhibit measures to promote gender equality, including a critical mass of women, role models, diverse leaders and inclusive organisational cultures. This paper explores relationships between organisational justice and equality interventions to better understand gaps between equality policies and practices using a comparative case study approach in a male-dominated sector. A combination of questionnaire and interview data analysis with employees in three case organisations in the construction sector are used to outline links between perceptions of gender equality initiatives and organisational justice, and the mechanisms used to reinforce in-group dominance. The findings culminate in the development of an Employee Alignment Model and a discussion of how this relates to the organisational climate for gender equality work. The findings suggest that the development of interactional organisational justice is an important precursor for successful gender equality interventions in organisations. These findings have implications for those looking to minimize unintentional harm of policies or interventions to improve gender equality.

## Introduction

The imperative to address gender inequalities has been established as a key social justice issue; this is of particular relevance in the context of the United Kingdom construction industry, where women made up only 14.3% of the construction workforce in 2021 (Office for National Statistics, 2021). Data from the Construction Industry Training Board (CITB 2016) finds the underrepresentation of women in construction is more severe in some roles such as site supervisors 0.7%, site managers 0.9%, engineering professionals 9.8% and notably the trades, where women represent less than 1% of the workforce (Bridges et al., 2020). However, greater representation of women is found within administrative and clerical support roles 87.6%, sales and customer services 63.7% and legal professionals and associates 50%. The difference of representation in roles that are often viewed as traditionally masculine is apparent.

There are also challenges when considering senior representation, with women making up only 3.1% of contracts managers and 12.6% of Architects (CITB 2016), roles recognised to be senior within the construction sector. The data suggests that there is horizontal and vertical segregation of women within the construction sector alongside barriers to entry.An explanation for the underrepresentation of women can be found in the challenges they face in the construction sector. Gender inequality was identified by Navarro-Astor et al.’s (2017) systematic review, covering 15 years of research, as consistent and multi-level (structural, cultural and personal). Including structural constraints such as inflexible working practices that undermine a return to work after maternity leave (Aboagye-Nimo et al., 2019) and discriminatory behaviour by employees and managers (Galea et al., 2020).

When motivated to address gender inequality, the construction sector favours equality policies, diversity training, and client-led, project-based solutions, such as initiatives to encourage more women and underrepresented ethnic minority groups into industry (Galea et al., 2015). However, such approaches are often episodic, rarely measured, and are predominantly focused on entry-level recruitment (Barnard et al., 2010) and short-term outcomes (Chadney, 2006), often resulting in little meaningful change (Clarke et al., 2017). While some companies offer a more comprehensive approach to equality, including equality action plans and awards, there is no robust evidence to demonstrate impact.

A focus on attracting women into the sector in numerical terms, rather than addressing inequality holistically (Bagilhole, 2009), hinders progress and may contribute to unintended outcomes (Van den Brink and Benschop, 2012; Powell et al., 2010; Navarro-Astor et al., 2017). Further, equality policies, training and other initiatives based on presumed agreement on the need for action (Ness, 2011) are unlikely to gain cooperation from a workforce that may be hostile or dismissive to minorities. More broadly, critical diversity studies acknowledge these power relations (Gotsis and Kortezi, 2014) and the limitations of diversity networks to offer support to employees (Dennissen et al., 2020) or training to combat discrimination (Noon, 2018).

Outside of construction political groundswell has led to the development and implementation of a raft of measures to try to address gender inequalities. These include broad-reaching programmes based on principles of gender mainstreaming (Stratigaki, 2005; Verloo, 2005), a focus on representation through gender disaggregated data analysis and gender budgeting (Morley, 2007), collection of gender pay gap data (O’Reilly et al., 2015), to organisationally-based gender equality plans (Van den Brink and Stobbe, 2014) and individual-level initiatives to support career development (Kossek et al., 2016; de Oliveira et al., 2019). Establishing efficacy of gender equality actions is notoriously difficult given the complexity of macro, meso and micro-level variables that contribute to gendered effects. Moreover, there are significant challenges with translation of equality policies into changes in practice, often fueled by lack of resources, strategic direction and priority (Das and Parker, 1999; Lee et al., 2010; Samuelson et al., 2019), indicating a policy-practice gap.

It is further argued that attempts to counter gender inequalities are fraught with risks of unintended consequences (Caffrey et al., 2016; Leslie 2019) and potential harms that exacerbate the problem or cause backlash (Smithson and Stokoe, 2005).

*Backfire* affects the intended outcome negatively, impacting both in- and out-groups, where the in-group is defined as those with access to power and resource and the out-group is defined as those with restricted access to power and resource (Allport et al., 1979). Examples of backfire can be seen in the following literature. Set up a simulated hiring environment and found that white men applying for roles within a pro-diversity company expressed more concern about being discriminated against. The white men made an inadequate impression in interviews relative to other white men applying to a diversity neutral company. Sanchez and Medkik (2004) found that although trainees initially reacted positively to diversity training, they held more differential views towards out-group members after attending the training. In the first of two studies around race and gender, Brown et al. (2000) found women who believed they were appointed due to gender performed worse than women who thought they were randomly selected or selected on merit. In the second study, Brown et al. (2000) concluded that beliefs held by students about benefiting from affirmative action in college submissions affected their grade point average. These examples of backfire demonstrate the potential for both in-group and out-group harm.

*Negative spillover* is where an unintended outcome is impacted in an unintended direction. Examples of negative spillover are illustrated in a study by Dover et al. (2016) involving 135 participants; it was discovered that whites and some Latinos were more likely to view an organisation with a diversity award as fair and respectful. However, participants were also more likely to derogate any discrimination claimant in award-winning companies. Additionally, Plaut et al. (2011) conducted a five study investigation into the reactions of dominant group members. They found “whites” had lower levels of support for multiculturalism than “racial minorities” and did not perceive multiculturalism to be inclusive. Negative spillover has also been termed backlash (Faludi, 1991) and can be employed to serve a political and economic purpose.

*Positive spillover* is where the Company Equality Approach (CEA) affect an unintended outcome in a desirable direction, usually impacting non-targets attitudes and perceptions. Seen in the study by Williams and Bauer (1994) that found that in- and out-group participants rated a fictitious company more favourably when managing diversity controls were included in the brochure.

*False progress* is where the intended outcome is impacted, and the direction is desirable, but real change is not created. Examples of false progress were seen in the construction literature around Heathrow and the Olympic Park, where targets for women employed in construction roles were used to demonstrate progress and legacy (Clarke and Gribling, 2008). However, these appointments were often short-term non-construction roles, so they failed to impact long-term change (Minnaert, 2014).

When considered through the lens of unintended consequences, attitudes and behaviours of in-group managers and peers (employees) have sufficient impacts on out-group members to warrant separate investigation (Navarro-Astor et al., 2017; Leslie, 2019). Especially as we consider differential possession of power and resources between in-group and out-group members (Allport et al., 1979). This paper focuses on behavioural factors and how these are linked to employee attitudes. The key contribution of this paper is the exploration of organisational conditions, perceptions of organisational justice and how these relate to attitudes towards actions to address inequalities, conceptualised in an employee alignment model.

## Gender Equality Actions and Impacts

In response to gender inequalities, including vertical and horizontal segregation, various approaches have been taken that have resulted in policy-interventions at macro, meso and micro levels. For example, in Higher Education and research institutions in the United Kingdom, the Athena SWAN Charter was introduced in 2005 to combat gender inequalities in STEM disciplines, and since 2015 across the arts, humanities, and social sciences. This initiative has seen high levels of engagement and is attributed to some levels of success in addressing some of the key barriers for women (Barnard, 2017; Rosser et al., 2019). However, Caffrey et al.’s (2016) evaluation of Athena SWAN demonstrates unintended consequences, including reproduction of gender inequalities, suggesting a mixed picture at best.

When considering the experiences of women in construction, it is important to note the experience of multiple complex forms of hostility and discrimination where identity intersects more than one site of disadvantage, which is highlighted in the concept of intersectionality (Crenshaw, 1990; McCall, 2005; Powell and Sang, 2013). For example, a black woman may identify with her ethnicity and gender alongside her job role, familial status and class (Carbado et al., 2013) together, these produce particular experiences in social relations. Introduced initially to illustrate the exclusion of black women from mainstream feminist thinking, intersectionality has been adopted as a way of understanding the complexity of different identities more generally (Carbado et al., 2013) and criticised as being reappropriated with white narratives (Dhamoon, 2011). However, the flexibility and ambiguity of the concept undermine “its” systematic application in methodological approaches (Martinez Dy et al., 2014). Therefore, as the focus of this study is on establishing a more general picture of relations between groups of people in organisations, it is based on the idea that experiential differences between in-groups and out-groups are greater than within out-groups (Brewer, 1999).

Although research makes recommendations for policy and practice, it stops short of scrutinising those recommendations for outcomes and unintended consequences (Leslie, 2019). Therefore, even in specific contexts like the construction sector, the impact of equality actions is unclear. Furthermore, the lack of empirical research on understanding the implications of company equality approaches on all and subsets of employees means that it is difficult to determine their effects (Clarke et al., 2017), including any unintended outcomes of equality work (Leslie, 2019). There has, however, been research on organisational climate for diversity (Hickes-Clarke and Iles, 2000; Schneider et al., 1994), which is linked to a focus on organisational culture (Burke and McKeen, 1992; Moran and Volkwien, 1992). The advantage of this perspective is that it acknowledges broad contextual factors that may help or hinder efforts to tackle inequalities. As Hickes-Clarke and Iles (2000: 326) put it: “for managing diversity to be successful, the organisation needs to develop a positive climate for “diversity,” which includes recognition for the need for diversity, support for diversity, organisational justice and perceptions of policy support. Importantly part of the definition of diversity climate includes perception of organisational justice in HR policies, thereby indicating linkages between efficacy of diversity actions and perceptions of broader organisational practices, which also underpins the Employee Alignment Model developed in this paper.

## Conceptualising Organisational Justice

From a social-psychological perspective, definitions of organisational justice foreground perceptions of fairness in the workplace (Byrne and Cropanzano, 2001; Colquitt et al., 2013). It has been found that low levels of perceived injustice in construction companies are institutional rather than cultural, in that they relate to organisations, not the overall industry, reinforcing the idea that perceptions of justice are formed by the actions of the organisation (Loosemore and Teck-Heng Lin, 2016). This is likely to play out in a plethora of fora and working relationships in which “fairness” is expressed and experienced over time. What is clear here is that subjective experiences underpin our understanding of such a concept. Research that operationalises concepts of organisational justice is primarily concerned with understanding individual experiences and perceptions (Colquitt et al., 2005) and potential consequences. For example, Crawshaw et al. (2013) found that perceptions of organisational justice are positively related to performance and wellbeing at work–i.e., the fairer the organisation is perceived to be the higher levels of performance and wellbeing are indicated. Response to broader questions regarding the role of organisational culture in behaviour have argued for a justice climate (Goldberg et al., 2011) as a specific component of culture. As the field of organisational justice has developed, integrated justice as a concept points to the ways that people form perceptions of justice (Colquitt et al., 2005), the relationship between group acceptance and organisational justice (Lind and Tyler 1988) and how early perceptions of justice form a lens through which employees view the organisation (Tyler and Lind 1992).

Altogether, concepts of organisational justice make distinctions between what are termed distributive, procedural and interactional justice (Colquitt et al., 2005), culminating in a “three-factor model” (Miller et al., 2012). Distributive justice encompasses “justice in the distribution of rewards and costs between ‘persons’ (Homans, 1974, 1961: 74); “perceived fairness of the outcomes or allocations that an individual “receives” (Folger and Cropanzano, 1998: xxi); and of fair “distribution” (Leventhal, 1980: 4). Hence, Distributive Justice (DJ) is concerned with fair, transparent sharing of available resources. In organisations this includes employee benefits such as wages, pensions, bonuses; but also, resources that can facilitate successful fulfilment of role, including training and development. Here gender inequalities might be identified in a gender pay gap. An important aspect of distributive justice is that judgements of justice are the product of comparisons between individual experiences and perceptions of allocation of resources across the group, in line with the idea of relative deprivation (Stouffer et al., 1949; Cropanzano and Ambrose, 2015). Consequently, we can understand distributive justice in organisations to be subjective and crucially influenced by the context in which people work. However, the focus on “how things “are” and not “how they came to “be” means that a distributive justice perspective misses some of the nuances of organisational decision-making (Leventhal, 1980) and lacks detail on the comparative aspect of employee perceptions (Colquitt et al., 2005). In response to this Procedural Justice (PJ) foregrounds the process of resource allocation in how fairness is recognized. Definitions of procedural justice include an “individuals” belief that allocative procedures which satisfy certain criteria are fair and “appropriate” (Leventhal, 1980: 30); further concerns about the fairness of the decision-making procedures used to determine “outcomes” (Bobocel and Holmvall, 2001: 86). This perspective on gender inequalities focuses on the process of pay decisions rather than the outcomes of those decisions that may result in a gender pay gap, for example. In research distributive and procedural justice are shown to positively relate to job satisfaction, evaluation of supervisor, conflict/harmony, trust in management and negatively relate to turnover intentions (Alexander and Ruderman, 1987) and increase positive attitudes and behaviours for organisational change (Shah, 2011). In fact, positive perceptions of procedural justice–which includes trust, consistency, ethics–can compensate for more negative perceptions of distributive justice (Thibaut and Walker, 1975). However, the focus on distribution of resources and the procedures that underpin decision-making–material and bureaucratic perspectives on fairness–sidestep how decision-making comes about through relationships between people and how it is communicated in interactions.

The concept of Interactional Justice (IJ) places communications and relations between people at the centre, which includes rather top-down, hierarchical ideas about agents and receivers (Bies, 1986) and “concerns about the quality of interpersonal treatment that they receive during the enactment of decision “procedures” (Bobocel and Holmvall, 2001: 86). More broadly, interactional justice is focused on the style in which information is communicated, opportunities for involvement in decision making and having a voice in the workplace (Mowbray et al., 2015). Coming back to the gender pay gap example, this might encompass in-depth consultation exercises with employees and union representatives around decision-making on pay and the adoption of a management approach that facilitates discussion of any concerns. In relation to interactional justice, Greenberg (1993) made further distinctions between: respect, propriety and concern regarding distributive outcomes, named interpersonal justice; and truthfulness, justification and concern for people regarding knowledge of procedures, termed informational justice; resulting in an organisational justice four-factor model (Colquitt et al., 2013). This distinction gives a finer-grained perspective on the role that communication and interaction plays in how employees feel about their workplace. For example, Fuchs and Edwards (2012) found that only interpersonal justice perceptions play a significant role in predicting pro-change behaviour. The four-factor model is argued to be the most appropriate to operationalise in research on relatively contained actions or single initiatives (Miller et al., 2012), for example in exploring employee perceptions of 360-degree appraisal programme. However, when taking a more holistic view of organisations as a complex interface between multiple actions the three-factor model is deemed more appropriate (Miller et al., 2012) as it provides a clearer framework to apply systematically across complex datasets.

Clearly there are some overlaps between the concepts of distributive, procedural and international justice when applied in practice (Stone-Romero and Stone, 2005), for example at what point does process become communication of process and distinct from the process itself? How can we define processes as separate from social relations rather than an expression of it? However, in trying to unpack organisational justice as a concept, distinctions can assist in uncovering the complex relationships between what goes on in organisations and how employees feel about it. Masterson et al. (2000) suggest that the application of distributive, procedural and interactional justice concepts can aid understanding of organisational practices at the meso and macro levels. Consequently, we argue that organisational justice concepts can be used as a tool in the analysis of gender equality action and impacts in organisations. Exploring employee Perceptions of Organisational Justice (POJ) at distributive, procedural and interactional levels can map out equality action as a terrain and the differential roles and efficacy of different actors: human resources may play a key role in the establishment and maintenance of fair processes, with managers being more influential regarding distribution of resources and communication of policies and practices.

Parallel studies had suggested that a relationship between the two variables might exist (Roberson and Stevens, 2006; Brooke and Tyler, 2010; Fujimoto, 2013; Kulik and Yiqiong, 2015; Dahanayake et al., 2018). Roberson and Stevens (2006) found a relationship between justice perceptions and equality incidents; specifically, that equality incidents that had been viewed as negative were more likely to cite justice issues. Stone-Romero and Stone (2005) theorized that employee perceptions of justice underpinned employee response to equality approaches, proposing that a perceived absence of justice would result in deviant behaviours toward the out-group such as harassment and discrimination. A relationship between the two variables, employee attitudes toward company equality approaches and perceptions of interactional justice is implied but lacking in empirical evidence (see also Roberson and Stevens, 2006; Brooke and Tyler, 2010; Fujimoto, 2013; Kulik and Yiqiong, 2015; Dahanayake et al., 2018).

However, inside and outside of construction CDS has identified challenges when implementing CEA that have lead to inconsistent outcomes, policy practice-gaps (Lee et al., 2010; Samuelson et al., 2019) and unintended consequences of equality approaches (Leslie, 2019) suggesting that a radical reexamination equality practice is required.

The inconsistent outcomes of CEA suggest a sociological element, the research therefore focuses on overall employees and utilises Organisational Justice as a potential theory that could explain the inconsistency of CEA through employee attitudes and resulting resistance to CEA.

To research the relationship between organisational justice and perceptions of equality actions in construction sector companies the following research questions guided the empirical data collection:

RQ1: What are the intentions, motivations and defining characteristics of the case company equality approaches?

RQ2: Is there a relationship between employee perceptions of justice and employee attitudes toward company equality approaches?

RQ3: What are the consequences of the relationship between perceptions of justice and employee attitudes towards company equality approaches?

## Methods

The research undertook a pragmatic position to establish the nature of the relationship between employee Perceptions of Organisational Justice (POJ) and employee Attitudes toward company Equality Approaches (AEA) before conducting an in-depth exploration of the consequences of that relationship within the context of the three case companies and their associated equality approaches.

As indicated in the research questions, the aim of the research is to explore the relationship between POJ and AEA across three companies in the same industry (main contractor companies in construction). There was an average of 20% of women across the organisations, 45% of employees working on site, with senior managers making up an average of 13% of workers (Table 1).

**TABLE 1:** Population and sample for three case studies.

Characteristic	Category	Company frequency						Total
		Org A		Org B		Org C		
		*N*	%	*N*	%	*N*	%	
Population	N/A	341		500		2,500		3,341
Sample	N/A	165	48	218	44	399	16	782
Gender	Male	131	71	172	72	324	73	627
	Female	42	23	49	20	97	22	188
	Other	1	1	2	1	0	0	3

To meet the aim and respond to the research questions a case study methodology was developed (Yin, 2014). Case study methodology provides a flexible framework within which different research methods can be employed, enabling the research to respond to the research questions adequately (Yin, 2014), which can be interpreted as a qualitative endeavour (Yazan, 2015), or one in which quantitative approaches are prioritised (Elman et al., 2016). Through an exploration of relationships between “employee”s POJ and AEA the motivations, strategy and initiatives that make up the CEA are analysed in relationship to employee perceptions and their experiences in the workplace. (EDI practitioners), based in the case study organisation, and interviews and surveys with employees. First steps in the case study were the interviews with experts that focused on the CEA. Appropriately worded questions (Gillham, 2005) were prepared for expert interviews focused on the following areas: the “expert”s role and responsibility in the business; the specific undertaking of the company equality approach; motivations, intent, priorities and implementation of the company equality approach. Expert interviews were analysed using the Dass and Parker framework (1999) to categorise and understand what is intended by the company equality approaches taken in each case. The framework encompasses diversity perspectives, pressures for and against diversity, strategic responses and implementation. In doing so it offers a multifaceted and in-depth tool that systematically analyses CEA including an account of organisational intent missing in other models (Yadav and Lenka, 2020). This is an important aspect to consider as the CEA′ intent and motivation could affect how employees perceive and respond to it.

Second, the survey with employees collected data on perceptions of justice (Moorman, 1991), AEA and attitudes to equality more broadly (Ng and Burke (2004). The survey was distributed to employees by an internal contact in the Case Study who sent general invitations to participate. The percentage of women participants, employees working on-site and senior managers were representative of the Case Study population. Response rates from each case were Case A 54, Case B 48 and Case C 18%. Given the larger size of case C (A, N = 341, B, N = 500, C, N = 2,500) the response rates were deemed acceptable Field (2014). Quantitative data was analysed using regression analysis and the Kruskal-Wallis test to interpret employee questionnaire responses to questions regarding the variables, perceptions of justice (DJ, PJ, IJ), Personal Attitude toward Equality (PAE) and AEA. Third, interviews with employees explored in-depth the experiences of employees using Critical Incident Technique (CIT) to focus participants on attitudes and behaviours to events that have occurred, which is a more reliable measure than asking participants how they think they would act to imagined scenarios (Flanagan, 1954; Butterfield et al., 2005). Process, *in vivo* and descriptive coding (Saldaña, 2021), were used to create a rich understanding of the qualitative data, combined with insights from open questionnaire questions by employing pattern coding techniques to develop overarching categories, clusters and themes. This approach enabled a more in-depth exploration of some of the quantitative findings and revealed nuance in the employee experience (Table 2).

**TABLE 2:** Survey and interview participants by case.

	Case A	Case B	Case C	Total
Expert interviews	1	1	1	3
Surveys with employees	165	218	399	782
Interviews with employees	1	5	2	8

The data from each stage of the case study methodology were triangulated to build a picture of relationships between organisational justice and equality actions and initiatives as they are perceived and experienced by employees. The cross-case analysis applied the Kruskal-Wallis test to appreciate Case to case differences. Finally, axial coding was used to analyse the combined qualitative and quantitative data as it is recognised as the most effective tool to combine fractured data (Saldaña, 2021); resulting in the development of the theoretical framework and model of employee alignment.

The ethical evaluation was generated through an Ethical Approval Risk Assessment (EARA). The EARA considered all points of the research that might impact on human participants or cases. Informed consent was of particular importance and participants were made aware that their participation would be anonymous, the purpose of the study, their right to drop out, and how to complain about the way the study was conducted. Anonymity was maintained by asking participants to self select for the interviews as part of the questionnaire, those who put themselves forward were not revealed to the organisation and were given a code to which they were referred to. All cases were allocated a case letter (A,B or C) which was not revealed to each case to provide organisational, as well as individual anonymity. After establishing the risks and proposing mitigation methods, the EARA achieved ethical approval from Loughborough ABCE ethics committee.

## Findings

### Perceptions of Gender Equality Initiatives and Organisational Justice

The quantitative questionnaire data collected across the three cases allows for the analysis of relationships between POJ and ʻAEA. The individual case regression analysis established a relationship between the interactional justice (IJ) measure and AEA by testing the hypothesis There is a relationship between AEA and Perceptions of 1) Distributive Justice (DJ); 2) Procedural Justice (PJ); and 3) Interactional Justice (IJ). In all three cases a substantial relationship was determined by IJ having a significance of less than 0.05 (Case A 0.002, Case B 0.000, Case C 0.000) (See Tables 3–8).

**TABLE 3:** Case A: H2. Regression model summary.

Model	R	R square	Adjusted R square	Std. Error of the estimate	*F*
1	0.417^a^	0.174	0.158	0.66943	11.288

a Predictors: (Constant), IJ, DJ, PJ.

**Hypothesis 1** (A) There is a relationship between employee Attitudes towards Equality Approaches (AEA) and Perceptions of (a) Distributive Justice (DJ); (b) Procedural Justice (PJ); and (c) Interactional Justice (IJ).

**TABLE 4:** Case A: H2. Coefficients.

Model	Unstandardised coefficients	Standardised coefficients		
	B	Std. error	Beta	t	Sig	
1	(Constant)	2.700	0.366	0.087	7.372	0.000
	DJ	0.061	0.059	0.070	1.029	0.305
	PJ	0.057	0.075	0.316	0.761	0.448
	IJ	0.339	0.106		3.197	0.002

Dependent Variable: AEA, Independent Variable DJ, PJ, IJ.

**TABLE 5:** Case B: H2. Regression model summary.

Model	R	R square	Adjusted R square	Std. Error of the estimate	*F*
1	0.295^a^	0.087	0.074	1.07813	6.776

a Predictors: (Constant), IJ, DJ, PJ.

**TABLE 6:** Case B: H2. Coefficients.

Model	Unstandardized coefficients		Standardized coefficients
	B	Std. Error	Beta	*t*	Sig	
1	(Constant)	2.008	0.431		4.660	0.000
	DJ	0.092	0.065	0.093	1.417	0.158
	PJ	0.039	0.090	0.030	0.436	0.664
	IJ	0.326	0.083	0.270	3.947	0.000

Dependent variable: AEA.

**TABLE 7:** Case C: H2. Regression model summary.

Model	R	R square	Adjusted R square	Std. Error of the estimate	*F*
1	0.524^a^	0.274	0.269	0.54965	45.144

a Predictors: (Constant), IJ, DJ, PJ.

**TABLE 8:** Case C: H2. Coefficients.

Model	Unstandardized coefficients		Standardized coefficients
	B	Std. Error	Beta	*t*	Sig	
1	(Constant)	2.862	0.180		15.898	0.000
	DJ	0.046	0.033	0.069	1.403	0.161
	PJ	0.277	0.041	0.373	6.806	0.000
	IJ	0.134	0.046	0.162	2.904	0.004

Dependent Variable: AEA.

There were some differences between the cases, in the quantitative analysis the overall POJ and AEA scores for Case B were found to differ from those of Cases A and C, suggesting that Case B had a lower POJ that impacted upon AEA. As Case Bs PAE was not significantly different from that of Case A or C reinforced the idea that AEA can be influenced by the Case. The other main difference in the case study data was that overtly hostile behaviours were only present in Case B, of which there were three incidents and three questionnaire responses (QRs). Examples of hostility to out-group members were not present in Case A and C, despite a range of QRs being mentioned by participants. Therefore, the data analysis points to key areas of variance between the cases (Table 9).

**TABLE 9:** Cross case comparison using Mann-Whitney.

Factor	Between cases		*U*	*z*	*p*	*r*
DJ	Case A	Case B	14,662.00	3.107	0.002	−0.16
	Case B	Case C	54.594	5.29	0.000	0.212
	Case A	Case C	35.21	1.32	0.188	0.06
PJ	Case A	Case B	83.99	−9.06	0.000	−0.46
	Case B	Case C	70.49	12.79	0.000	0.51
	Case A	Case C	36,423	2.00	0.045	0.51
IJ	Case A	Case B	180.461	−13.39	0.000	−0.68
	Case B	Case C	76.98	15.90	0.000	0.64
	Case A	Case C	33,121.00	0.117	0.907	0.049
PAE	Case A	Case B	0.799	0.272	0.671	0.01
	Case B	Case C	44.69	0.57	0.57	0.02
	Case A	Case C	34.389	0.839	0.402	0.035
AEA	Case A	Case B	111.720	−10.531	0.000	−0.54
	Case B	Case C	72.18	13.60	0.000	0.55
	Case A	Case C	34,030.50	0.637	0.524	0.027

*U* indicates the test statistic, *z* indicates the standardised test statistic, *p* indicates probability and *r* indicates the effect size estimate. Effect size estimates above 0.3 are considered to be medium, above 0.5 are considered to be large.

**Hypothesis 2** The distribution of (a) DJ; (b) PJ; (c) IJ; (d) PAE; and (e) AEA is not the same across MCCs.

Analysis of the interview data with regard to beliefs held by employees about the company context, the equality approach and perceptions of in-groups and out-groups enables a deeper understanding of relationships found in the quantitative analysis. IJ was also found to correlate with AEA in all three of the cases in the interviews, this was evidenced in the interviews by low POJ participants consistent concerns regarding positive discrimination and fears that they had been overlooked for promotion due to Company Equality Approaches (CEA). As described by one white male low POJ participant:

One of the things I disagree with is turning around and saying “we”ve got ten posts and five of them have got to be women. That would be my only gripe with it, they tend to be pushing women in there for the sake of it A1.1.1

Across the three cases, there was evidence that employees did not perceive the “company”s equality approach the way that those implementing them had intended. Initiatives were set up in the cases as linked to company strategies to varying degrees. The expert interviews found that Case B was clear that initiatives were driven by the client, but recognised internal need to address equality was developing. Cases A and B both stated that their company equality approaches were tied into the overall company strategy, however the Dass and Parker framework (1999) was used to analyse the responses and found that, in practice, all initiatives were episodic and lacked consistent evaluation, demonstrating a policy-practice gap.

The motivations of the case organisations focused on client need and internal demand. However, “employees” perceptions of motivating factors were less likely to mirror the organisational motivation and more likely to be in line with personal values or perceptions of justice. For example, low perception of justice participants stated societal or legislative factors as responsible for motivating the company equality approach. Whereas employees with mid to high perceptions of justice were more likely to state business benefits as a motivating factor. In particular, there were concerns raised about “positive” “discrimination” despite none of the companies adopting measures that would be objectively described as such. Therefore, the research shows a trend towards misunderstanding and misinterpretation of gender equality approaches, which appear to be linked to broader employee perceptions of organisational justice in those contexts.

The individual case analysis and cross-case analysis identified attitudes and behaviours that were predominant, or only apparent in respondents with low POJ (Table 3). Table 3 demonstrates that the low POJ category groups all exhibited between five and six of these behaviours. The Mid/high POJ category groups exhibited between zero and two of these behaviours. The difference in attitude and behaviour of the low POJ category participants and the mid/high POJ category participants implies that these attitudes are linked to low POJ.

### Mechanisms That Reinforce In-Group Dominance

Mechanisms that support the in-group were found in all case study companies. Common mechanisms included arguing against positive action, benevolent inequality (often expressed most clearly as protective paternalism) and identifying outliers–the bad apple defence, and these were evident in all three cases. Discussions of inequalities frequently turned to concerns about in-group members being overlooked and an aversion to out-group members being supported or promoted because of their identity rather than capability was strongly argued in all cases. Furthermore, perceptions that out-group members were receiving unjust reward resulted in the in-group claiming that out-groups were less committed and capable. As one white male participant put it:

“We need to ensure that we don’t turn positive efforts to get minorities and women into Construction, into a crusade. If they are keen and capable, then assist and support to succeed. However, “let”s not shove people from minorities into our industry, if they are less than committed, and potentially commit reverse discrimination on our historic sector demographic” (QR.A14)

Another white male participant talked about their experiences: "I can and have had minorities chosen over myself due to that and not their ability or drive, that then makes me wonder if that is a positive thing” (QR.A13) Concerns about commitment, ability and drive of out-group members suggest that out-group members are judged and positioned as tokens, undermining meritocracy. Linked to this, narratives about out-group capabilities highlight how out-group members are othered by the in-group under the guise of inclusivity. One white male low POJ participant frames women in the construction sector as needing to prove their worth, more so than their in-group colleagues, meaning their competence requires repeated visible demonstration to be accepted by the in-group:

“Positives outcomes is, it is going to give more women the opportunities to demonstrate what they can or “can”t do. As I said before ‘I’ve worked for some fantastic female engineers, one thing I do notice about them though is that all of them, well not all of them, but most of them have been much keener and much more enthusiastic about what they are doing, but as a rule, ‘you’re getting first-class engineers. And I think that is; I think that will give girls, er women the opportunity to come through and prove themselves”. (A1.1.3)

When “participant”s accepted that out-group members experience discrimination, it was described as isolated incidents or perpetrated by individuals–the “bad “apple” defence - and therefore, broad actions to address the issues are not deemed appropriate. Minimising the impact of discrimination on out-group members and individualising the discriminatory behaviour feeds into a narrative that actions to address inequalities promote positive discrimination and are harmful to the organisation.

Other mechanisms that were found in one or two of the cases but not all three, include suggesting that discriminatory behaviours are unintentional, normalising problematic behaviour, hostility towards the out-group, denial of a problem, inequalities being understandable, downplaying out-group experiences in comparison to in-group issues, and championing the in-group. Normalising was evidenced in an incident outlined by a white male, high POJ participant that saw the introduction of different positions of power:

“On my last job, one of the general foremen is an old guy. And he often made sexist or racist jokes. I “don”t believe he was racist, but I think he thought it was OK to make them. I probably should have said something, but I “didn”t because he is quite senior”. (B4.4)

Here tensions can be identified - although the participant expressed that he was uncomfortable with the comments and deemed them to be inappropriate, he did not challenge them due to the seniority of the foreman. Furthermore, the participant went on to defend the foreman as not being racist, as he did not believe the foreman would treat the ground workers differently from “*traditional white British people.*” In this way, he positions problematic in-group behaviour without consequence.

Importantly many of the in-group supporting mechanisms were found to go unchallenged and even supported by in and out-group members, suggesting that mechanisms are embedded in organisational structures and cultures; enabling actions that support the in-group to be accepted. However, as we have made fine-grained differentiation between the mechanisms at play, it is important to recognise the interaction and overlap between these mechanisms which suggests a harmonious melody, despite being played on different instruments. Together these mechanisms represent an impressive range of responses when faced with inequalities that work to undermine actions towards social justice.

### Employee Alignment Model

**FIGURE 1:**
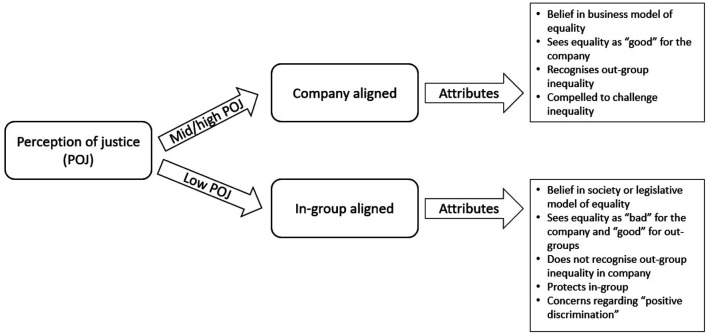
Employee alignment model.

Triangulation of the organisational strategic intent to equality approaches, employee perceptions of equality, company equality approaches and organisational justice, and outcome stages of the research enables the evolution of the Employee Alignment Model. Underlying the model are the key differences identified in the case study data in relation to perceptions of organisational justice: low levels of justice map onto those predominantly aligned to the in-group and mid-high levels of justice map onto those predominantly aligned to the company (Figure 1; Table 10).

**TABLE 10:** Outcomes of in-group and company alignment.

Axial category	Perception of justice	Outcomes
Company-aligned	High or medium employee perception of justice	Inclusive Narrative—Out-group support is good for the business
		Attitude: Support for equality, balanced view on equality approaches
		Desire to hold unequal behaviour to account
		Embraces company equality approach. Learning mindset–seeks to improve their treatment of out-groups
In-group-aligned	Low employee perception of justice	Divisive Narrative–In-group support is good for business. Out-group support is good for out-group individuals
		Attitude: Spurious support of equality
		Justifies inappropriate in-group behaviour towards out-groups
		Resists company equality approach. Seeks to undermine the need for company equality approach

In-group aligned employees exhibit spurious support of company equality approaches, perceive out-group members as a threat and employ in-group supporting mechanisms. This position reinforces discrimination and bias as acceptable, legitimating fellow in-group aligned members, which in turn feeds into discriminatory organisational cultures. This position also creates friction and weakens working and personal relationships with company aligned employees. Crucially for those wishing to address inequalities is the negative impacts this position has on out-group members, including retention, productivity and satisfaction at work.

In contrast, the company aligned group members genuinely support equality approaches, perceive benefits to the company and express supportive and inclusive behaviours. This position allows for the holding to account of discriminatory behaviours of colleagues and produces an organisational culture that frames discriminatory behaviour as bad for business. This position reinforces equality as an opportunity for learning and improvement of individual management and team working skills for company aligned employees. A more positive context is developed for out-group employees, resulting in improved out-group retention, teamworking and workplace satisfaction.

## Discussion

The study confirms the hypothesis that there is a positive relationship between employee perceptions of interactional justice and employee attitudes toward company equality approaches; specifically, employee attitudes toward company equality approach were more supportive when employees perceived interactional justice. The relationship suggests that perceptions of interactional justice influence “employees” attitudes to company approaches, so if employees perceived interactions with their peers and managers as unjust they were less likely to hold positive attitudes towards the work the company is undertaking regarding equality. Where attitudes towards company equality approaches were more negative, employees demonstrated resistance and employed in-group supporting mechanisms. Therefore, improving perceptions of interactional justice could hold the key to improving employee attitudes towards, and efficacy of, company equality approaches and employee behaviours towards out-groups.

A reoccurring challenge to progression around equality is the failure to account for employee response to equality approaches (Kalev et al., 2006; Van den Brink and Benschop, 2012). Although the case experts noted that there were employees who were resistant to equality approaches, consideration was absent regarding how the resistant employees would respond to equality approaches and if any unintended consequences would arise. Only one Case company (Case C) recognised that equality approaches could have unintended consequences. Despite this, they had no strategy to measure or contain those outcomes.

Employee resistance to equality approaches was noted in the responses to the interviews and questionnaire; examples include denial of the existence of out-group inequality, rejection of poster campaigns by employees on-site and obstruction and rejection of training around equality (Sanchez and Medkik, 2004; Plaut et al., 2011; Kaiser et al., 2013; Major and Kaiser, 2017). These overt displays of resistance were supported by more covert actions, including frequent concerns regarding “positive discrimination” and roles being taken from “qualified employees.” The problem is therefore framed as an issue of meritocracy: concerns regarding the need for objective employment structures when employing out-group individuals, may be better understood as an in-group protection mechanism based on assumptions that out-group individuals are “less” capable and committed than their in-group counterparts (Allport, 1979; Brewer, 1999; Dovidio et al., 2005). Resistance is therefore framed as being about competence and experience—“the right person for the “job”–when viewed in the context of subjective judgments made of “colleagues” performance, reasonable objections take on a rather different character. Therefore, this study reiterates others who have found that resistance from employees is likely to have contributed to company equality initiatives failing to eradicate inequality (Kalev et al., 2006; Coetzee and Bezuidenhout, 2011; Major and Kaiser, 2017).

This study found the main factor in employee perceptions of CEA to be their alignment status. In-group aligned employees consistently raised concerns regarding positive discrimination, whereas company aligned employees did not. Employee resistance to equality approaches does not only impede the achievement of successful outcomes of the company equality approach; there can also be grave implications for the out-group. The fostering of equality approaches on a resistant audience has been found to result in a backlash against the out-group (Faludi, 1991; Kalev et al., 2006), assumptions that the problem of inequality has been solved resulting in the rejection of discrimination claims (Kaiser et al., 2013) and other unintended consequences (Leslie, 2019). The study findings that demonstrated discrimination towards the out-group highlight the impact of resistance to CEA and out-groups generally. It is important to note that a failure to adequately consider employee resistance to any company equality approach or initiative may result in out-groups experiencing more inequality and discrimination (Leslie, 2019).

Conversely company-aligned employees reported impetus to challenge inequality when it arose, a willingness to educate themselves on matters of equality and a desire to work for an organisation that actively supported out-groups. It is plausible that company-aligned employees fall into a virtuous circle, and in-group aligned employees fall into a vicious circle (Masuch, 1985). Identifying and understanding these patterns of behaviour in an EDI context, starting with employee POJ, is vital in address out-group inequality.

The Employee alignment model suggests that general employee perceptions of justice must be identified and addressed before implementing any CEA. Where negative perceptions of justice are evident organsiations should focus on improving these perceptions of fairness to reduce instances of backlash or negative spillover. Where positive perceptions of justice are identified a more direct implementation of equality approaches may be appropriate. Once employee perceive their organisations as just, work tackling inequality and improving equality is likely to have impact and less likely to produce unintended consequences.

### Future Work

The exploratory nature of the research has opened the door to much potential future work that has real world application regarding reducing inequality and unintended consequences of equality approaches. Understanding more about the relationship between in-group-aligned and company-aligned employees is crucial to reducing unintended consequences and their impact upon out-groups. Future research and practice could benefit from establishing indications regarding the percentage of company-group-aligned employees that are required to reduce in-group supportive behaviours and to be evidence of reducing out-group inequality in the workplace.

Extending the study beyond large main contractor firms to determine if perceptions of justice impacts employee attitudes towards company equality approaches in other organisation types, sectors. Additionally, exploring the relationship between perceptions of justice and equality in other contexts; for example, it is equally plausible that the challenges around justice applied to companies may also impact on social groups. The relationships between austerity and rising reports of hate crime toward out-groups suggest justice and equality may be linked in any social settings.

There could also be benefit in establishing the implications regarding impact of AEA on POJ, are in-groups, out-groups, in-group aligned or company aligned individuals POJ impacted by their AEA. Establishing if the presence of vicious and virtuous circles could add weight to the Employee alignment model.

The construction focus was borne of the researcher’s lived experience, but it should be noted that the exploratory work did not find consistent solutions to inequality in the broader research around equality or diversity management, suggesting that the Employee alignment model and a move towards behavioural equality, meaning equality approaches that consider how people behave in their organisational contexts, not simply how we want them to behave, could provide might provide much needed insight for the future trajectory of equality work.

### Limitations

The research investigates the relationship between POJ and AEA, and generates a theory regarding associated behaviours, as evidenced in the employee alignment model. Due to the exploratory approach undertaken, a limitation of the work is that the model has not been validated, future work by the researchers intends to test the model in differing contexts including higher education and the public sector.

There was a gap of a year between data gathering by questionnaire and employee interviews within each case. This reduced questionnaire respondent availability for interview, creating recruitment issues and resulting in eight interviews with employees across the three cases (A = 1, B = 5, C = 2). The planned number of interviews had been three per case, meaning the case-by-case analysis was biased as Case B had more respondents. Had there been a more proportional representation of participants across the three cases, the research may have provided more insights into each case.

The case companies all operated within public sector frameworks meaning that, if instructed by the public sector client, they were liable to meet the Public Sector Equality Duties (PSED). Therefore, the cases presented work in a slightly different context than private contractors and companies outside of construction who do not have to respond to these legislative requirements. This may affect the study’s generalisability when considering the impact of CEA. However, since the CEA was found to have minimal impact on the employee alignment model, it is posited that the model could is relevant to the construction industry and wider organisations generally, although further research should be undertaken to test this proposition.

## Conclusion

This paper utilised a multiple case study methodology that triangulated questionnaires, interviews, and desktop research to build a picture of the relationship between perceptions of justice and attitudes to equality approaches. Perceptions of justice and in particular Interactional Justice had a positive relationship with employee attitudes toward company equality approaches across all three cases. This suggests that employee perceptions of justice regarding managerial interactions were most likely to impact upon employee attitudes toward company equality approaches.

An Employee Alignment Model is put forward based on the findings of the qualitative analysis which found that alignment of employees, corresponded to organisational justice and perceptions of equality actions. It is suggested that employee perceptions of justice are crucial to understanding resistance to company equality approaches and backlash toward out-groups.

These findings are relevant to practitioners and organisations. First, they suggest creating a company working environment that is perceived as “just” is likely to reduce hostility and discrimination towards out-group members significantly. Second, the findings suggest that implementing equality approaches in unjust environments may increase hostile and discriminatory behaviour towards out-group members and this should be considered when designing equality policies (Jacobs and O’Neill, 2003; Caplan and Gilham, 2005; Christensen, 2013; McCarthy et al., 2013; Cross et al., 2017; McCarthy et al., 2019; Elomäki et al., 2020).

## Data Availability

The raw data supporting the conclusions of this article will be made available by the authors, without undue reservation.
